# Zika virus infection of pregnant rats and associated neurological consequences in the offspring

**DOI:** 10.1371/journal.pone.0218539

**Published:** 2019-06-20

**Authors:** Morgan L. Sherer, Pragyan Khanal, Gwen Talham, Erin M. Brannick, Mark S. Parcells, Jaclyn M. Schwarz

**Affiliations:** 1 University of Delaware, Department of Psychological and Brain Sciences, Newark, Delaware, United States of America; 2 University of Delaware, Office of Laboratory Animal Medicine, Newark, Delaware, United States of America; 3 University of Delaware, Department of Animal and Food Sciences, Newark, Delaware, United States of America; University of Modena and Reggio Emilia, ITALY

## Abstract

Zika virus (ZIKV) is a mosquito-borne flavivirus associated with microcephaly and other neurological disorders in infants born to infected mothers. Despite being declared an international emergency by the World Health Organization, very little is known about the mechanisms of ZIKV pathogenesis or the long-term consequences of maternal ZIKV infection in the affected offspring, largely due to the lack of appropriate rodent models. To address this issue, our lab has developed a working model of prenatal ZIKV infection in rats. In this study, we infected immune competent pregnant female rats with 10^5^–10^7^ PFU of ZIKV (PRVABC59, Puerto Rico/Human/Dec 2015) in order to examine its pathogenesis in the dams and pups. We examined the febrile response and sickness behavior in the dams, in addition to neonatal mortality, microglia morphology, cortical organization, apoptosis, and brain region-specific volumes in the offspring. Here, we demonstrate that pregnant and non-pregnant female rats have a distinct febrile response to ZIKV infection. Moreover, prenatal ZIKV infection increased cell death and reduced tissue volume in the hippocampus and cortex in the neonatal offspring. For the first time, we demonstrate the efficacy and validity of an immunocompetent rat model for maternal ZIKV infection that results in significant brain malformations in the neonatal offspring.

## Introduction

Zika virus (ZIKV) is a mosquito-borne flavivirus that was first isolated in 1947 from a rhesus macaque in Uganda [1]. ZIKV remained primarily a primate pathogen for decades, sporadically infecting humans until 2007, when it caused its first noteworthy human epidemic on the Yap islands in Micronesia, and then in 2013 when it reached New Caledonia, French Polynesia, and more extensively, Brazil in 2015 [2]. Since then, active transmission of ZIKV has been reported in over 70 countries and territories globally (http://www.cdc.gov/zika/geo/index.html). ZIKV infection during pregnancy is associated with an increased incidence of congenital malformations in the developing fetus, most notably, microcephaly. Through January 26th of 2016, 10,441 suspected and 2,366 confirmed ZIKV-associated microcephaly cases were reported in Brazil alone, prompting the World Health Organization to name ZIKV as a public health emergency of international concern [3].

Since its emergence, there has been a notable increase in research dedicated to understanding the pathogenicity of ZIKV in order to develop vaccines and therapeutic strategies. Despite these efforts, no effective therapies currently exist. This is partially due to limitations in current ZIKV animal models used for experimentation, which do not accurately mimic ZIKV infectivity in immunocompetent humans. The most widely used animal models for ZIKV infection utilize immunocompromised mice, and/or use atypical routes of infection such as: intracranial, intraperitoneal, and intravenous routes (see [4] and [5], for reviews). These models are necessary to examine ZIKV infection in a mouse because adult wild-type (WT) C57BL/6, Swiss Webster, BALB/c, and CD-1 mice are unable to sustain ZIKV infection following typical peripheral administration (subcutaneous, intra-peritoneal, and intravenous) [6–9].

Previous data from our lab demonstrate that rats are naturally immunocompromised during pregnancy, particularly during late gestation, a phenomenon that is also well-known in humans, resulting in altered immune responses and disease pathogenesis in pregnant women [10,11]. As a result, we hypothesized that pregnant rats may be naturally vulnerable to ZIKV infection, thus making them an ideal animal model for human maternal ZIKV infection. Here, we developed a rat model to study the effects of ZIKV infection during pregnancy, in order to examine its effects on the developing fetal brain.

In Experiment 1, we inoculated pregnant and non-pregnant female rats subcutaneously with three doses of ZIKV (ZIKV 1×10^5^, 1×10^6^, and 1×10^7^ PFU/dose) and assessed signs of infection in the females including body temperature, sickness behavior, changes in food and water consumption, as well as maternal and neonatal mortality. In Experiment 2, we assessed the impact of our highest dose of ZIKV (1×10^7^) on neonatal brain volume, cortical organization, viral presence, and apoptosis, as well as microglial gene expression, number, and morphology. This is the first study of its kind to demonstrate that subcutaneous infection of ZIKV in an immune competent pregnant female rat results in vertical transmission to the fetus, neural cell death, and associated brain malformations in the offspring.

## Materials and methods

### Animals and breeding

Adult male and female Sprague Dawley rats (Envigo Laboratories, Indianapolis, IN) were housed in same sex pairs in clear, polypropylene cages (45cm x 20.5cm x 24cm) with *ad libitum* access to food and water. The colony room was maintained at 22°C under a 12:12 hour light:dark cycle. Sixty nulliparous females aged 54–56 days in age were paired individually with male rats of the same age for breeding to generate litters in various cohorts throughout the course of the experiments. The presence of a vaginal plug was checked daily in order to confirm pregnancy and the date of conception, embryonic day 0 (E0). Day of birth (DOB, approximately E23) was assigned as Postnatal day 0 (P0). Male breeders were removed from cages and females were moved to a Biosafety Level 2 (BSL 2) animal isolation facility and were individually housed in clean cages prior to infection of the pregnant females on E18. All experiments were approved by the University of Delaware Institutional Animal Care and Use Committee (Animal Use Protocol #1306).

### ZIKV growth conditions and ZIKV inoculation

Vero C1008 cells (ATCC CRL-1586 TM) were grown in Dulbecco’s Minimum Essential Medium (DMEM), high glucose with 10% fetal bovine serum, 4mM L-glutamine, 1% penicillin/streptomycin/neomycin (PSN), and 0.5% fungizone and incubated at 37°C with 5% CO_2_. Human Puerto Rico (Dec. 2015) Zika Virus (ZIKV, strain PRVABC59, ATCC VR-1843, GenBank Accession: KU501215) was diluted 1:5, 1:10, 1:100, and 1:1000 in base growth medium without serum and plated onto PBS-washed Vero Cells in T25 flasks at 75% confluency. ZIKV and cells were incubated at 37°C for 1 hour to allow for adhesion and attachment, with growth medium added and observed for six days. Media from flasks displaying over 80% of ZIKV-associated cytopathic effects (CPE; cell rounding, syncytia, granularity, plaque formation) was collected and cellular debris removed by centrifugation at 500 x *g* for 10 minutes. ZIKV stocks were divided into aliquots, frozen, and stored at -80°C prior to titrating using 10-fold dilutions (10^−1^–10^−9^) 10-fold dilutions of virus were plated, in triplicate, across (2) 12-well dishes (10^−1^–10^−8^), incubated for 72–96 hrs, then fixed with 4% paraformaldehyde for 1 hr. Infected cells were washed with 1X PBS and stained with human anti-ZIKA (Kerafast human anti-ZIKA) followed by goat anti-human IgG Alexa 568, washed, and nucleic acids were stained with 3 nM DAPI in 1X PBS, 10% glycerol. ZIKA plaques were counted by IFA and mean titers determined for each stock. This work was carried out under IBC protocol # 16–021.

Pregnant and non-pregnant females were inoculated subcutaneously around E18 with either a diluent control (0.1 ml of the same culture media used to grow ZIKV) or ZIKV (doses ranging from 1×10^5^–10^7^ PFU in 0.1 ml culture media). These doses were selected based on previously published studies that use these doses in mouse models [9,12–15]; [Pregnant Diluent, n = 8; Non-pregnant Diluent, n = 9; Pregnant ZIKV 10^5^, n = 10; Non-pregnant ZIKV10^5^, n = 5; Pregnant ZIKV 10^6^, n = 4; Non-pregnant ZIKV 10^6^, n = 6; Pregnant ZIKV 10^7^, n = 13; Non-pregnant ZIKV 10^7^, n = 5].

Infection on E18 of gestation was selected for the current study based on previous work in our lab indicating that during late gestation, the immune system of the pregnant female is significantly compromised and therefore unable to respond effectively to an immune challenge (10). Moreover, late gestation (E18-23) in rats is a time point during which significant brain growth occurs, similar to the second trimester equivalent in humans [16]. Rats were inoculated on E18 based on the presence of a sperm plug on E1; however, after the rats gave birth, we estimate that dams were inoculated between E17 and E19. These minor variations in the date of inoculation did not significantly impact the main effect of ZIKV infection on cell death or brain volumes that were measured in the affected offspring across the various litters (see Results Experiment 2).

### Experiment 1: Monitoring the symptoms of ZIKV infection in pregnant and non-pregnant female rats

Experiment 1 aimed to assess the symptoms of infection following various doses of ZIKV infection (1×10^5^–10^7^ PFU/dose). The febrile response, sickness behavior, food and water consumption, and pup mortality were monitored daily for 5–7 days post-inoculation (dpi) in all dams. Daily rat body temperature was measured each evening, between 18:00 and 20:00 hours using a rectal probe thermometer. Sickness behavior including shivering, curled up posture, and piloerection [17] was also recorded at these time points. Food and water bottles were weighed each evening in order to determine how much was consumed in the previous 24 hours. At the time of birth, and over the subsequent two days prior to neonatal brain tissue collection for the next experiment, any pup deaths were recorded.

### Experiment 2: Examination of the impact of prenatal ZIKV infection on tissue volume, cell death, and microglia in the hippocampus and cortex of neonatal offspring

#### Tissue collection

The sex of the pups was noted on P2 based on anogenital distance. The pups were euthanized by rapid decapitation. Based on the data obtained in Experiment 1, which suggested very few differences in the maternal response to infection across the various doses, Experiment 2 focused on comparing differences between the pup brains from offspring that had received the highest ZIKV dose (1×10^7^) to our diluent control offspring. Two males and two females from each litter of these groups were selected for tissue collection, and thus multiple litters are represented across each treatment group. Pup brains were collected and immediately fixed in 4% ice-cold paraformaldehyde (PFA) for 24 hours at 4°C. These brains were then submerged into 10% gelatin solution, transferred to fresh 4% PFA, followed by a 30% sucrose solution, and then a fresh 30% sucrose solution, each in 24-hour intervals at 4°C.

An additional male and female from each group were selected for PCR analysis. Following perfusion, half of each brain was extracted for hippocampus/cortex using the guide of a rat atlas and immediately flash frozen in cold isopentane and stored at -80°C until ready for processing. The hemisphere collected for either analysis was randomized.

#### Immunohistochemistry

Pup brains were sliced at 20 μm using a Leica cryostat set to -25°C and immediately thaw-mounted onto Superfrost++ Micro Slides (VWR). The slides were stored at 4°C. The target protein chosen for staining was Ionized calcium-binding adaptor molecule 1 (Iba1), a protein expressed specifically in microglia [18]. Slides were washed with 0.01M phosphate-buffered saline (PBS) 3 times, for 5 minutes each, and then incubated for 1 hour with a blocking buffer solution containing PBS with 5% normal goat serum (Vector Laboratories), 0.3% Triton X (Fisher Scientific), and 0.03% H_2_O_2_. Slides were washed again with PBS and incubated with 150 μL of primary antibody (rabbit, anti-Iba1, 1:5000; Wako Chemicals, Richmond, VA) overnight at room temperature. Slides were washed again with PBS the next day and incubated with 150 μL of a biotinylated secondary antibody (goat, anti-rabbit IgG, 1:1000; Jackson ImmunoResearch Laboratories, West Grove, PA, USA) for 2 hours at room temperature. Slides underwent another wash with PBS and were incubated for 2 hours with avidin-biotin complex (ABC) Solution (Vectastain ABC kit; Cat. No. PK6100 Standard; Vector Laboratories) to identify immunostaining. Slides were then washed and stained with cresyl violet for three minutes, before slides were rinsed, dehydrated, cover-slipped with Permount (Fisher Scientific), and stored at room temperature until analysis.

A separate set of pup brains (n = 4 animals per group) were sliced at 4 μm. Serial sections were stained with either Hematoxylin and eosin (H&E) (Fisher Scientific) to characterize neural pathology, anti-NeuN to label neurons (mouse, anti-NeuN IgG, 1:500; Sigma-Aldrich, St. Louis, MO), or with an anti-ZIKV antibody (rat, anti-ZIKV IgG, 1:100; Kerafast cat # EDW003) to determine viral presence. Fluorescent slides were incubated in secondary antibody (NeuN stain: Goat anti-mouse IgG, FITC, 1:500; ZIKV stain: Goat anti-rat IgG, Alexa 488, 1:50; ThermoFisher) and were coverslipped using DAPI mounting medium (VECTASHIELD Antifade Mounting Media, Vector Laboratories).

#### Image acquisition

For all experiments, confocal fluorescence images were acquired with the following: Zeiss LSM 780 microscope equipped with 488 and 633 lasers, using a 20x (0.75 NA) or 40x (1.2 NA) water-immersion objectives and Zeiss Zen software. H&E and Iba1 images were captured on a Zeiss Axio Imager M2 microscope using a 20x (0.75 NA) or 63x (1.4 NA) oil objective and StereoInvestigator software.

#### Real-time PCR

Messenger RNA (mRNA) was extracted from pup brains using Isol-RNA Lysis Reagent (Cat. No. 2302700, 5 PRIME). Extracted RNA (1000ng) was then subjected to DNase treatment to remove any genomic DNA prior to cDNA synthesis using the QuantiTect Reverse Transcription Kit (Cat. No. 205314, Qiagen). Relative gene expression was measured using the RealMasterMix Fast SYBR Kit (Cat. No. 2200830, 5 PRIME) in 10 μL reactions on a CFX96Touch real time PCR machine for the following genes: CD11b, P2Y12, MHCII, and CD68. These particular genes were chosen for analysis due to their involvement in microglial activation. These primers were ordered through Integrated DNA Technologies and diluted to a final concentration of 0.13 μM for the real-time PCR reaction. The sequences of primers were as follows: CD11b forward: CTGGGAGATGTGAATGGAG, reverse: ACTGATGCTGGCTACTGATG (NM_012711.1); P2Y12 forward: GATTCTTTCTGTTGCCATCT, reverse: CCGACTTCAAGAAAGAACAT (XM_006232408.3); MHCII forward: AGAGACCATCTGGAGACTTG, reverse: CATCTGGGGTGTTGTTGGA (KC222936.1); and CD68 forward: GCTTCTGTTGCGGAAATAC, reverse: AGATTGGTCACTGGCGCAA (NM_001031638.1). Rplp1 was used as the reference/housekeeping gene for all samples as it was not significantly different across experimental conditions (neither treatment nor sex). For each reaction, the quantitative threshold amplification cycle number (Cq) was determined, and the 2-ΔΔCq method was used to calculate the relative gene expression of each gene in question.

#### Stereological analysis of apoptosis, microglia cell counts, and tissue volume in the neonatal hippocampus and cortex

Using the Paxinos, Ashwell, and Tork Atlas of the Developing Rat Nervous System (Figures 169–181; 2^nd^ edition; 1994), five coronal brain slices, at approximately every other slice in the series, were selected from each rat for unbiased stereology. Within the StereoInvestigator software, the optical fractionator method [19,20] (Microbrightfield Inc., Williston, VT, USA) was used to count cresyl violet-stained apoptotic cells and Iba1-stained cells manually within each frame using a 63X oil objective lens, at an optical dissector height of 4 μm with a 1 μm guard zone both on top and below [21]. Contour tracing of individual hippocampus regions (dentate gyrus, CA1, and CA3) and cerebral cortex (CX, including predominantly the parietal cortex) were drawn manually around the outermost portion of each region in the left hemisphere.

Cresyl violet-stained cells were counted as apoptotic cells if they demonstrated characteristic intense staining and cellular condensation. Cells were counted as Iba1-positive if the entire, rounded cell body was visible (average diameter approx. 15–25 μm) and if the dark stain evenly permeated across the entire body of the cell. Based on shape and configuration of processes, Iba1-positive cells were classified into four morphological categories [22–24]. The four cell types include: 1) round/amoeboid microglia with no processes; 2) stout microglia with short processes; 3) thick microglia with thicker, longer processes; and 4) thin microglia with thinner, long, ramified processes. The total count for Iba1-positive cells within each category was taken for all sections of the dentate gyrus, CA1, CA3, and CX by an investigator blind to treatment condition associated with each tissue sample.

Volume estimates for each contour tracing were also obtained using Cavalieri’s principle within the StereoInvestigator program for each target region (dentate gyrus, CA1, CA3, and Cortex) and calculated by multiplying the area given by the Cavalier estimator for each contour by the thickness of the sample slice (20 μm) to obtain the estimated volume of each contour of a specific slice. Apoptosis, microglia morphology, and the total number of all microglia within the CA1, CA3, dentate gyrus, cortex, whole hippocampus, and total microglia were calculated as a function of volume per mm^3^.

### Experimental design and statistical analysis

**Fig 1:**
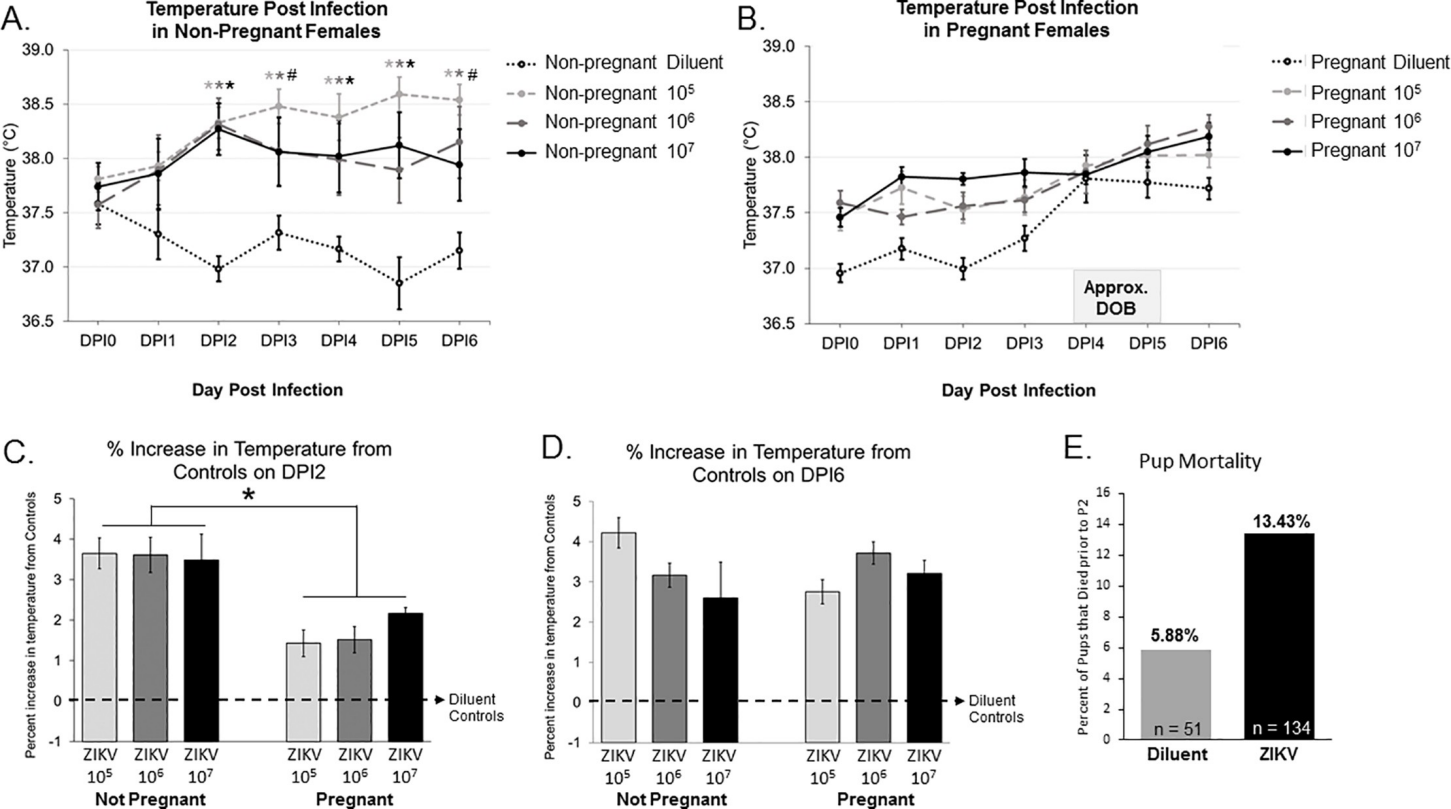
Examination of febrile response following ZIKV inoculation. (**A**) In non-pregnant females, there was a significant Treatment x Day interaction (F_12.141, 72.844_ = 4.378, p < 0.001). Post hoc comparisons revealed that by DPI2 all three non-pregnant ZIKV infected groups had a significantly higher temperature compared to the non-pregnant diluent treated groups (10^5^: P < 0.001; 10^6^: P < 0.001; 10^7^: P < 0.001), and that this effect persisted until six days following injection (DPI6) (10^5^: P = 0.001; 10^6^: P = 0.009; 10^7^: P = 0.068). (**B**) In the pregnant females, statistical analysis revealed a main effect of day (F_4.473, 134.176_ = 19.665, p < 0.001) and no interaction with ZIKV inoculation. (**C**) Percent increase in body temperature from diluent treated controls was analyzed on DPI2, and this analysis revealed a significant main effect of pregnancy condition on DPI2 (F_1,42_ = 38.07, P < 0.001). n = 4–13 animals per group. (**D**) Percent increase in body temperature from diluent treated controls was analyzed on DPI6, this analysis revealed no significant difference between groups. (**E**) Neonatal mortality was not significantly different between the diluent treated group 5.88% (n = 51) and the ZIKV group 13.43% (n = 134) (Χ^2^ = 2.093; p = 0.148). *: p < 0.05.

All data were analyzed using the statistical software program (SPSS; IBM). Febrile response as well as food and water consumption were analyzed in control and ZIKV-infected dams using repeated measures analysis of variance (ANOVA) with day post-inoculation as the within subject factor and ZIKV infection (dose) as the between-subjects factor. In these analyses, the Greenhouse-Geisser correction was used to correct for violating the assumption of sphericity with this particular repeated-measures ANOVA. Bonferroni -adjusted, pairwise comparisons were used for pair-wise post-hoc comparisons. A two-way ANOVA was used to compare percent increase in body temperature in ZIKV-inoculated pregnant and non-pregnant controls with pregnancy condition and ZIKV dose as factors. Neonatal mortality data was analyzed using a Chi-square test across the infected and diluent treated litters. Data for microglia morphology and the total number of all microglia within the CA1, CA3, dentate gyrus, cortex, whole hippocampus, and total microglia were calculated as a function of volume per mm^3^. Volume, apoptosis, gene expression, and microglial counts, and microglial morphology were analyzed across the two treatment groups (Diluent vs ZIKV 1×10^7^) using an unpaired two-tailed t-test. The accepted significance level for all analyses was p < 0.05, and the data in the graphs (except in Fig 1E) represent the mean ± SEM of the data.

## Results

### Experiment 1

#### Febrile response, sickness behavior, food and water consumption, and mortality following ZIKV infection

The febrile response, sickness behavior, food and water consumption, and mortality were monitored each evening following infection in pregnant and non-pregnant females. Analysis of body temperature in the non-pregnant females revealed a significant Treatment x Day interaction (F_12.141, 72.844_ = 4.378, p < 0.001, 2-way RM ANOVA; **Fig 1A**). *Post-hoc* comparisons revealed that by 2 days post-infection (DPI2), all three non-pregnant ZIKV infected groups had a significantly higher temperature compared to the non-pregnant, diluent-treated groups (10^5^: P < 0.001; 10^6^: P < 0.001; 10^7^: P < 0.001 compared to the uninfected controls). This effect of ZIKV infection persisted six days following inoculation (DPI6) (10^5^: P = 0.001; 10^6^: P = 0.009; 10^7^: P = 0.068 compared to the uninfected controls; **Fig 1A**). In contrast, this effect of ZIKV infection was not seen in the pregnant females, as statistical analysis revealed only a main effect of day on the body temperature (F_4.473, 134.176_ = 19.665, p < 0.001, 2-way RM ANOVA) and no interaction with ZIKV (**Fig 1B**). In particular, pregnant females showed a convergence in the average body temperature around the day of birth, regardless of treatment group. Data from non-pregnant and pregnant females were also analyzed as a percent increase in body temperature from the body temperature of diluent-treated females, and analyzed using a two-way ANOVA for pregnancy condition and ZIKV dose as factors. This analysis revealed a significant main effect of pregnancy condition (F_1,42_ = 38.07, P < 0.001; **Fig 1C**), such that non-pregnant females had a significantly higher change in body temperature by DPI2 compared to pregnant females; this significance goes away on DPI6 (**Fig 1D**). These temperature changes did not differ by ZIKV dose in either condition. These data support our previous understanding that pregnancy can significantly attenuate immune responses to infection (10, 11).

We saw no observable sickness behaviors (shivering, curled up posture, or piloerection) in either pregnant or non-pregnant ZIKV-infected rats. In addition, we found no significant main effect of day on the amount of food or water consumed throughout the 5-day period of observation (Food consumed: F_3.050, 67.105_ = 1.317, p = 0.254; Water consumed: F_1.106, 24.338_ = 1.574, p = 0.224, 2-way RM ANOVA) or interaction of day x treatment (Food consumed: F_15.251, 67.105_ = 1.407, p = 0.169; Water consumed: F_5.531, 24.338_ = 1.022, p = 0.188; 2-way RM ANOVA) There was, however, a significant main effect of pregnancy condition on the total amount of water consumed, with pregnant females drinking significantly more water than non-pregnant females (p = 0.026), however, there was no significant main effect of pregnancy on the total amount of food consumed during the 5 day monitoring period (p = 0.117). There were no adult female mortalities as a result of ZIKV infection; however neonatal mortalities did occur (determined as the number of dead pups found in the cage between P0-P2). Although there was no statistically significant difference between the pup mortality rates between control and treated litters, the mortality rate was approximately doubled for ZIKV treated pups: 13.42% (18 deaths out of 134 total ZIKV pups, including all three ZIKV doses) compared to untreated control pups: 5.88% (3 deaths out of 51 total diluent-treated pups), indicating a potentially biologically significant increase in pup mortality following ZIKV infection (Χ^2^ = 2.093; p = 0.148; **Fig 1E**).

### Experiment 2

#### Volume analysis

**Fig 2:**
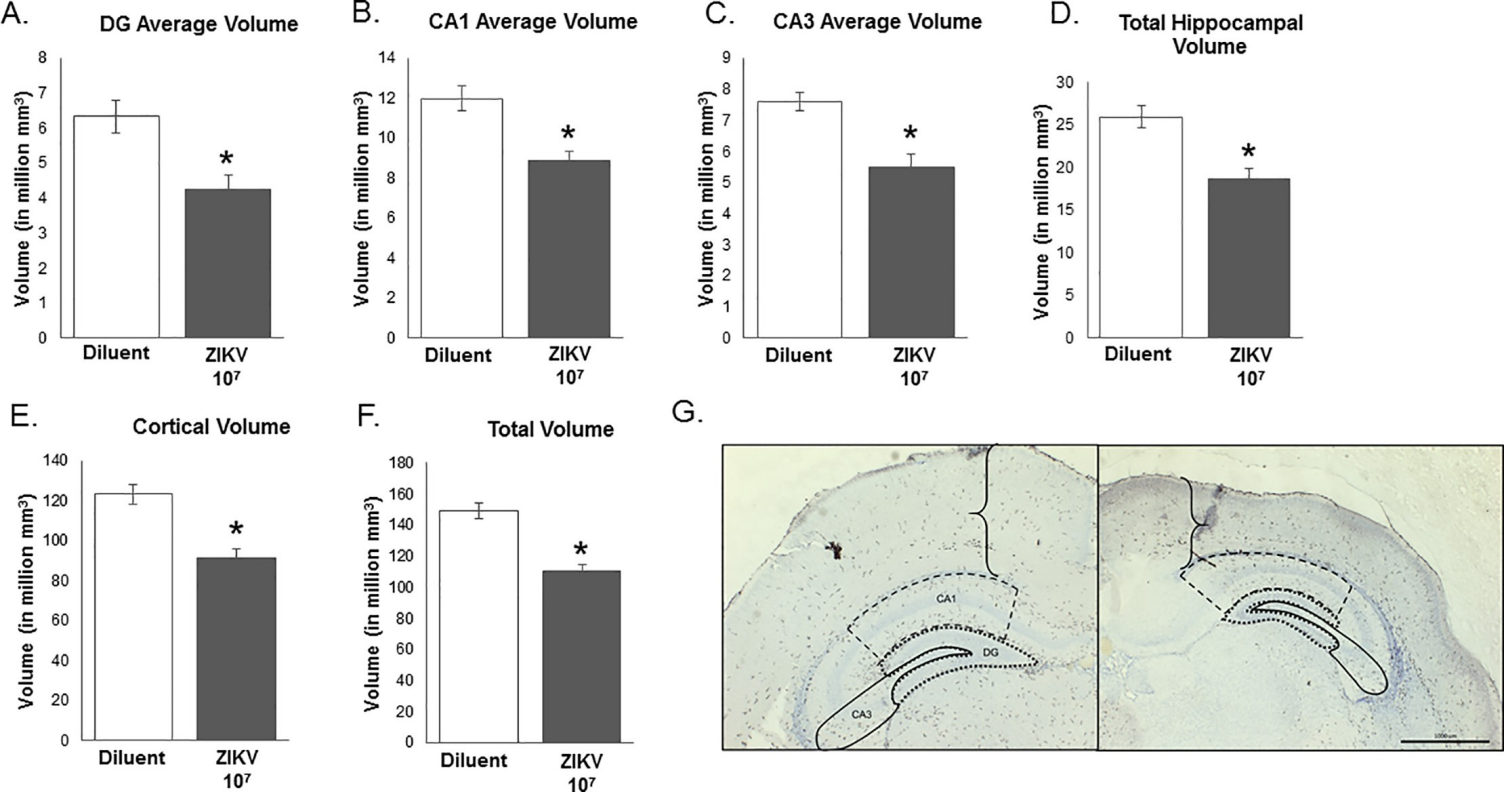
Analysis of hippocampal and cortical brain region volume in postnatal day 2 pups born of moms treated with ZIKV 10^7^ or diluent control. There was a significant effect of ZIKV infection on the volume of all brain regions examined (**A**, DG: F_1,10_ = 11.558, P = 0.007; **B**, CA1: F_1,10_ = 16.161, P = 0.002; **C**, CA3: F_1,10_ = 15.350, P = 0.003; **D**, Cortex: F_1,10_ = 22.282, P = 0.001; **E**, whole hippocampus: F_1,10_ = 16.380, P = 0.002; **F**, total volume: F_1,10_ = 33.137, P < 0.001), where brain volumes were significantly smaller in the pups infected with ZIKV compared to the uninfected controls. (**G**) Representative images of diluent and ZIKV pup cortex thickness and hippocampus size. n = 6 animals per group. *: p < 0.05.

Pup brains were collected from diluent control and 1×10^7^ ZIKV PFU-inoculated dams on postnatal day 2 (P2). The volume of the following brain regions was analyzed in the control and inoculated offspring, including the dentate gyrus, the CA1, and CA3 sub-regions of the hippocampus, the parietal cortex, as well as the whole hippocampus, and total hippocampal/cortex volumes. We found a significant effect of ZIKV infection on the volume of all brain regions examined (DG: F_1,10_ = 11.558, P = 0.007, **Fig 2A**; CA1: F_1,10_ = 16.161, P = 0.002, **Fig 2B**; CA3: F_1,10_ = 15.350, P = 0.003, **Fig 2C**; Cortex: F_1,10_ = 22.282, P = 0.001, **Fig 2D;** whole hippocampus: F_1,10_ = 16.380, P = 0.002, **Fig 2E**; total volume: F_1,10_ = 33.137, P < 0.001, **Fig 2F**); unpaired t-test), such that the volume of these brain regions were significantly smaller in the pups from ZIKV-injected females compared to the uninfected controls.

#### Cortical organization and viral presence

**Fig 3:**
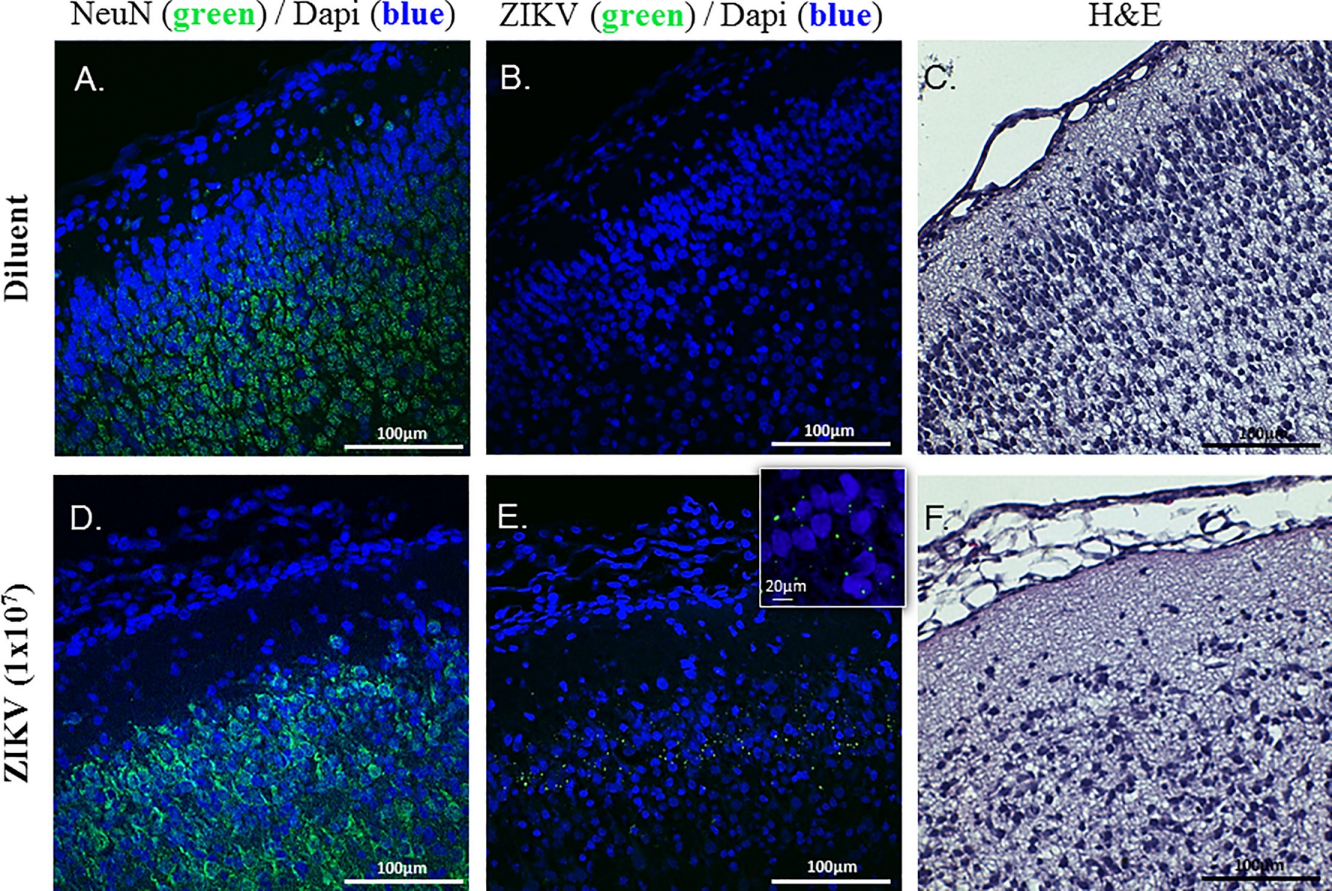
Histopathologic analysis of the pup brains. Hematoxylin and Eosin (H&E) staining (**C, F**) in conjunctions with anti-NeuN staining (NeuN: Green; DAPI: Blue; **A, D)** identified differences in neuronal arrangement and within the cerebral cortex of pups from ZIKV-treated dams, a finding that was indicative of cerebrocortical dysplasia. Anti-ZIKV staining identified viral presence in the ZIKV cortex with no positive staining found in the control animals (ZIKV: Green; DAPI: Blue; **B, E**). n = 4 animals per group. (20X magnification, scale bar = 100μm; higher magnification image on panel **E** was taken at 40X, scale bar = 20μm).

Histopathologic analysis of the pup brains (n = 4 animals per group) using Hematoxylin and Eosin (H&E) in conjunctions with anti-NeuN staining identified differences in neuronal arrangement within the cerebral cortex of pups from ZIKV-treated dams, a finding that was indicative of cerebrocortical dysplasia. Specifically, the ZIKV group appeared to have disorganized neuronal layering compared to controls. While the visible processes in the control group are primarily oriented perpendicular to the meninges, the ZIKV group has processes that are oriented parallel or oblique to the meninges, or are absent altogether (**Fig 3A–3F**). Further, anti-ZIKV staining identified viral presence in the cortex of the ZIKV+ pups with no positive staining found in the control pups. This finding suggests that vertical transmission is occurring from the dam to the developing fetus in this model, and that the virus is still detectable within the brain after 7 days post infection. Verification of these findings, however, will require additional quantitative analysis of neurons and viral presence.

#### Apoptosis analysis

**Fig 4:**
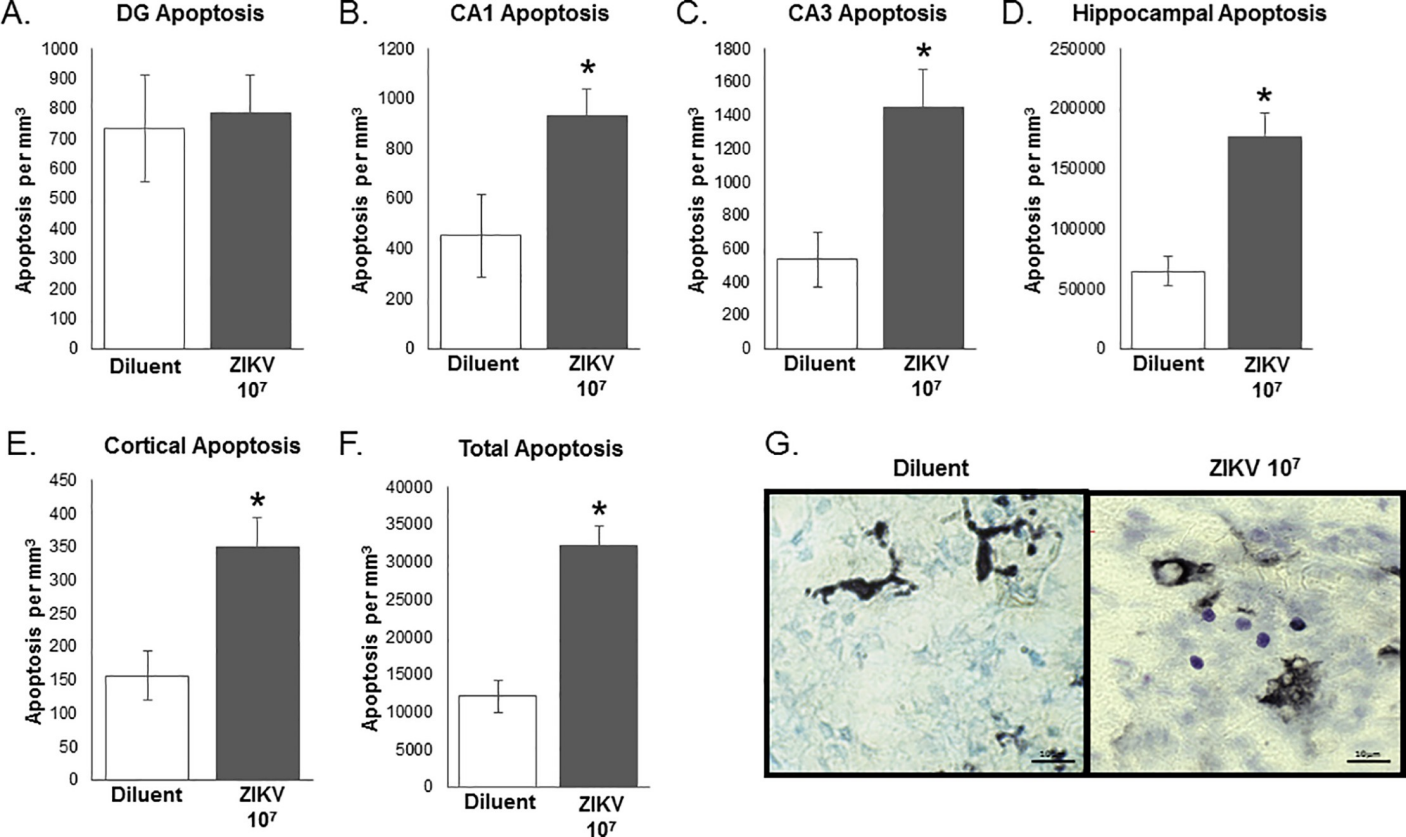
Assessment of apoptosis in hippocampal and cortical brain regions on postnatal day 2 in pups born of moms treated with ZIKV 10^7^ or diluent control. **(A)** There was no significant difference in apoptosis within the DG between ZIKV-infected and uninfected control pups (F_1,10_ = 0.053, P = 0.822). There was a significant increase in apoptosis per mm^3^ in the ZIKV-treated pups compared to the diluent control pups in the CA1 (**B**, F_1,10_ = 5.833, P = 0.036), CA3 (**C**, F_1,10_ = 10.784, P = 0.008), cortex (**D**, F_1,10_ = 11.813 P = 0.006), whole hippocampus (**E**, F_1,10_ = 21.102, P = 0.001) and the total apoptosis measured across all brain regions combined (**F**, F_1,10_ = 36.118, P < 0.001). (**G**) Representative images of cresyl violent stain in diluent pup and ZIKV pup brain, images taken in DG of the hippocampus. n = 6 animals per group. *: p < 0.05.

Apoptotic cell counts were analyzed as a function of the volume of each brain region (number of counted cells / total volume of brain region in mm^3^) in order to control for the differences in brain volume caused by maternal ZIKV inoculation (1×10^7^ ZIKV PFU). There was no significant difference in apoptosis within the DG between ZIKV and control pups (F_1,10_ = 0.053, P = 0.822, unpaired t-test; **Fig 4A**). There was, however, a significant increase in apoptosis per mm^3^ in the pups from ZIKV-infected dams compared to the diluent control pups in the CA1 (F_1,10_ = 5.833, P = 0.036, **Fig 4B**),CA3 (F_1,10_ = 10.784, P = 0.008, **Fig 4C**), Cortex (F_1,10_ = 11.813 P = 0.006, **Fig 4D**), whole hippocampus (F_1,10_ = 21.102, P = 0.001, **Fig 4E**). The total apoptosis counted across all brain regions combined was also significantly different as a result of ZIKV infection (F_1,10_ = 36.118, P < 0.001, **Fig 4F**).

#### Microglial counts and classification

**Fig 5:**
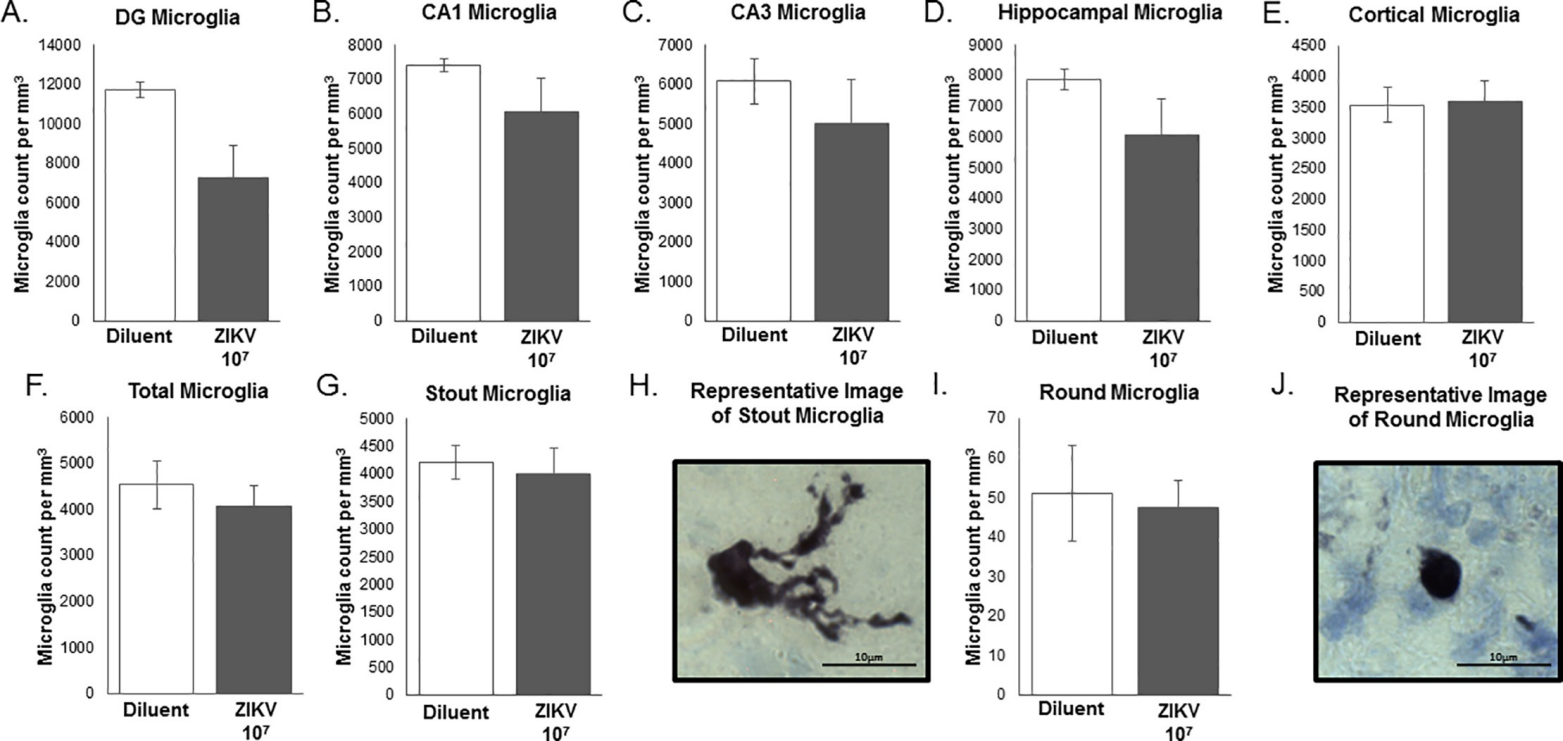
Investigation of microglial number and activation in hippocampal and cortical brain regions on postnatal day 2 in pups born of moms treated with ZIKV 10^7^ or diluent control. **(A)** There was a trend in the DG (F_1,10_ = 3.865, P = 0.078), indicating there are fewer microglia present in the ZIKV group compared to the diluent control group, though this effect was not significant. There was no significant effect of ZIKV infection on microglial numbers in (**B**) the CA1 of the hippocampus, (**C**) the CA3 of the hippocampus, (**D**) the hippocampus overall, (**E**) the cortex, (**F**) or in the analysis of all brain regions combined. Statistical analysis of microglial morphology revealed no significant difference in microglial morphology between treatment groups (**G**, Stout Microglia: F_1,10_ = 0.136, P = 0.720; **H**, Round Microglia: F_1,10_ = 0.067, P = 0.801). n = 6 animals per group. *: p < 0.05. (63X magnification, scale bar = 10μm).

Microglia are the primary immune cells of the brain that respond to infection, injury or cell death via an increase in number and a notable change in morphology. Microglial counts were analyzed as a function of volume (number of counted cells/total volume of brain region in mm^3^) in order to control for the differences in brain volume caused by maternal ZIKV inoculation. There is a trend in the DG (F_1,10_ = 3.865, P = 0.078, unpaired t-test, **Fig 5A**), indicating there were fewer microglia present in the ZIKV-treatment group compared to the diluent-treatment control group, although this effect was not significant. In all other brain regions, there were no significant effects of ZIKV infection on microglial number (**Fig 5B–5F**). We also classified the morphology of the microglia and found that on P2, in all brain regions examined, only stout and round morphologies were present, indicative of their relatively immature phenotype in the developing brain [18]. Statistical analysis revealed no significant difference in microglial morphology between treatment groups (Stout: F_1,10_ = 0.136, P = 0.720; Round: F_1,10_ = 0.067, P = 0.801, unpaired t-test; **Fig 5G and 5H**). (40X magnification, scale bar = 10μm).

#### Gene expression

**Fig 6:**
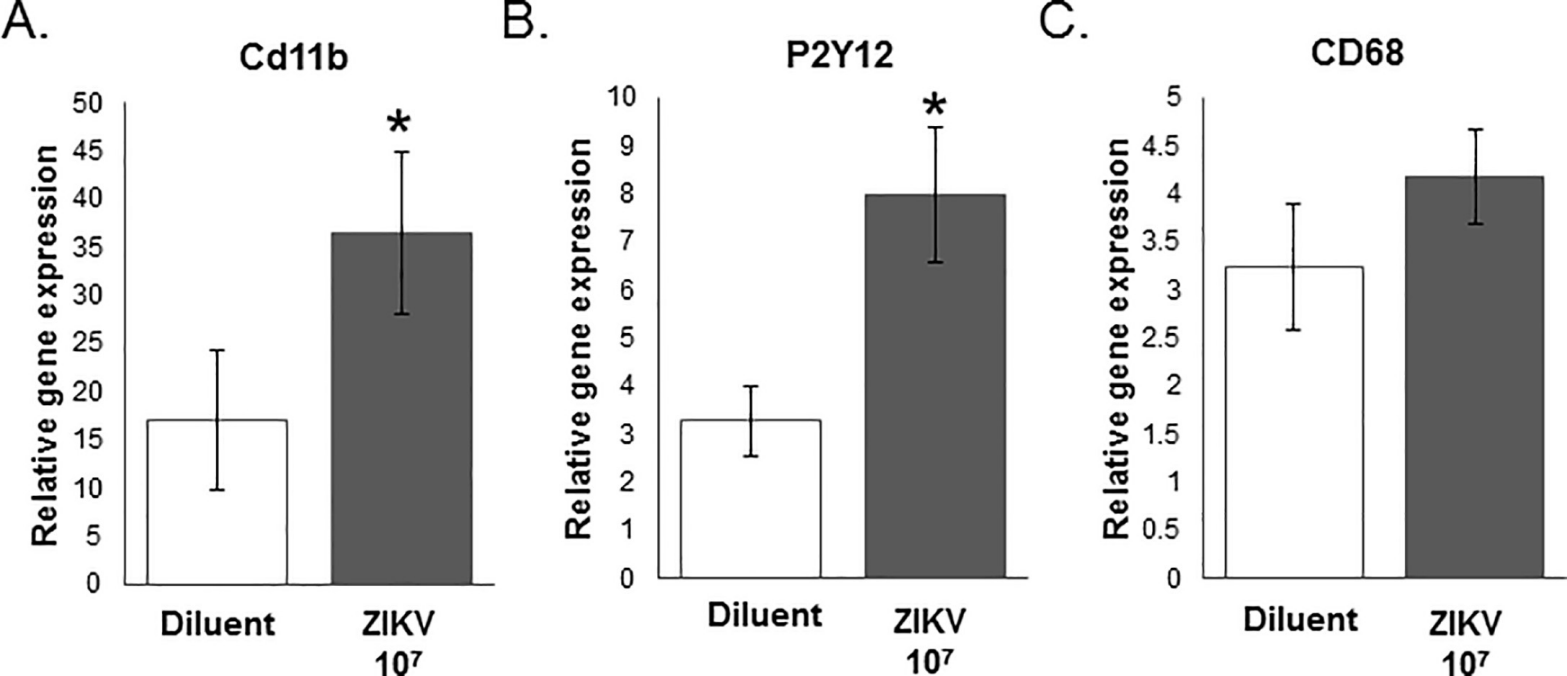
Gene expression analysis of microglial activation markers in the hippocampus/cortex of postnatal day 2 pups born of moms treated with ZIKV 10^7^ or diluent control. **(A)** CD11b gene expression was significantly higher in the hippocampus/cortex of P2 pups exposed to ZIKV during gestation compared to controls (F1,16 = -1.83, P = 0.042; d = 0.817053). (**B**) P2Y12 gene expression is also significantly higher in the ZIKV group compared to controls (F1,16 = -2.73, P = 0.007; d = 1.346703). (**C**) CD68 gene expression did not significantly differ between the ZIKV and diluent control group (F1,16 = -1.17, P = 0.130; d = 0.548408). n = 8 (Diluent) n = 10 (ZIKV) animals per group. *: p < 0.05.

Gene expression was analyzed from the hippocampus/cortex of P2 pups from mothers who were either exposed to ZIKV or a diluent control during gestation. We were interested in further exploring the effect of gestational exposure of ZIKV on microglial activation in the pup brain, therefore we measured genes associated with microglial activation: CD11b, P2Y12, MHCII, and CD68. CD11b is expressed by both resting and active microglia and forms part of complement receptor 3 that aids in the recognition and phagocytosis of antigens [25]. Here, we found that CD11b gene expression was significantly higher in the hippocampus/cortex of P2 pups exposed to ZIKV during gestation compared to controls (F_1,16_ = -1.83, P = 0.042; *d* = 0.817053, **Fig 6A**). P2Y12 is a metabotropic purinergic receptor exclusively expressed by microglia, which senses the release of ATP upon cellular injury, and is therefore a marker of microglial activation [26]. In this study we found that P2Y12 gene expression is also significantly higher in the ZIKV group compared to controls (F_1,16_ = -2.73, P = 0.007; *d* = 1.346703, **Fig 6B**). CD68 is a lysosomal protein expressed in high levels activated microglia and in low levels by resting microglia. We found that CD68 gene expression did not significantly differ between the ZIKV and diluent control group (F_1,16_ = -1.17, P = 0.130; *d* = 0.548408, **Fig 6C**). MHCII is expressed on the surface of antigen-presenting cells, primarily on microglia, and is responsible for antigen recognition and the activation of the adaptive immune system. MHCII gene expression levels were undetectable in both the ZIKV and control groups and were therefore not analyzed.

## Discussion

ZIKV is a teratogenic flavivirus that has been linked to a number of neurological malformations and complications in affected human offspring. Most notable of these is microcephaly, a neurological disorder characterized by a reduction in head circumference and brain cortical size [27]. Our results demonstrate that subcutaneous ZIKV injection of pregnant female rats is able to gain access to the fetal compartment and significantly impact the developing brain of the affected offspring, despite *minimal* symptoms in the dam. In particular, we demonstrate that maternal ZIKV inoculation results in increased cell death, reduced hippocampal and cortical volumes, and preliminary evidence of cereborcortical dysplasia in the neonatal brain. For the first time, we demonstrate the efficacy and validity of an immune competent rat model for maternal ZIKV infection that results in significant brain malformations in the affected offspring.

The majority of existing models for ZIKV infection have used immunosuppressed mice because adult mice can effectively mount an antiviral response that limits viremia [7]. In contrast, infection during pregnancy of female mice that lack type 1 interferon signaling produced a spectrum of congenital defects seen in some ZIKV-exposed infants [28], but these models lack generalizability to the human condition. Other models employ direct intracranial injection in immunocompetent mice to study the impact of ZIKV on the developing brain [29–33]. While these models provide valuable insight to the full effects of the relatively “unchecked” virus given the absence of a maternal interferon response, they lack the translational component, which is essential for understanding the complete pathophysiology of ZIKV infectivity. A previous study from our lab found that rats have a naturally suppressed immune system during pregnancy [10], making them a plausible candidate for a translational model of prenatal ZIKV infection. In the present study, we tested this hypothesis by injecting female rats subcutaneously, with the intent to mimic a mosquito bite—a common route of ZIKV infectivity in humans [7], and found that non-pregnant female rats mounted a significant febrile response to ZIKV compared to diluent controls. Interestingly, this effect of ZIKV did not occur in the pregnant female rats. The absence of a febrile response in the pregnant female rats suggests that the immune system of the pregnant dam does not *fully* respond to the viral infection (via pro-inflammatory cytokines, for instance), and thus further supports the notion of an immunosuppressed state during pregnancy [10–11]. It is important to note that ZIKV infection did not have an impact on overt sickness behavior or changes in food and water consumption in either group, suggesting that the ZIKV infected dams were not severely impacted by the virus, an effect that is similar to the symptoms described in ZIKV-infected humans [34,35], and furthermore, rules out any potential influence of maternal malnourishment on our observed pup outcomes.

Our results also demonstrate that pups born to ZIKV-inoculated mothers exhibited an increase in cell death in the hippocampus and cortex, a reduction in hippocampal and cortical volumes, and preliminary evidence of cerebrocortical dysplasia. The ability for a maternal ZIKV inoculation to produce these brain malformations and associated neural cell death in the affected pups suggests that the virus is not cleared by the maternal immune system, and was able disrupt normal brain development in the fetus.

The exact mechanism(s) by which the virus is able to cause this neurological disorder in our model remains unclear. In particular, the effects of ZIKV inoculation may be due to direct viral action in the developing brain via vertical transmission [1,36,37], or via an indirect, secondary mechanism such as maternal immune activation or cytokine production. Both ideas are plausible, but require further investigation. Maternal immune activation itself can produce aberrant neural development and behaviors in various animal models [36,38–41]; thus future experiments should investigate (1) the specific (if limited) immune response that pregnant female rats have to ZIKV inoculation, (2) how this response compares to the immune response produced in a non-pregnant females, and (3) which cytokines may be responsible for affecting neural development in the offspring. The results of these proposed future studies, which build directly from our findings here, would significantly inform our greater understanding of the mechanisms by which infection during pregnancy with viruses such as ZIKV, rubella, and cytomegalovirus can produce significant neurological effects in the offspring [37].

Microglia are the primary immune cells of the brain that express pattern recognition receptors dedicated to identifying and responding to viruses [42]. As a result, there are a number of studies that have examined microglia as a target of ZIKV and how microglia respond to ZIKV infection of other surrounding neural cells [43–45]. These animal models (i.e. mouse and human) have used immunohistochemistry to measure the impact of ZIKV exposure on microglia, they found that (1) microglia are able to be infected by ZIKV [45], (2) they can be activated following ZIKV infection [43–45], and (3) they exhibit altered coordination and organization following exposure to ZIKV (including microglial hyperplasia, perivascular cuffing etc.) [43]. Interestingly, we saw no change in total microglial number or microglial morphology between treatment groups, however subsequent quantitative RT-PCR analysis revealed increased expression of genes associated with microglial activation. Taken together, this suggests that microglial morphology may not be indicative in their function in this scenario, and that microglia are responsive to the viral challenge 7 days post inoculation. Thus, future studies could examine various, earlier time points post inoculation to fully understand the time course and the role of microglial cells in their response to maternal ZIKV during the early course of the infection.

In contrast to other brain regions examined, we found no detectable increase in apoptosis in the dentate gyrus of the offspring’s hippocampus, though we found a significant *decrease* in volume and a trending decrease in the number of microglia in this brain region because of maternal ZIKV infection. These data suggest that the dentate gyrus of the developing hippocampus may have a unique response to the viral infection relative to other regions in the developing brain. The dentate gyrus is one of only a few brain regions known for on-going neurogenesis throughout the lifespan [46]; thus, it is possible that this unique neural niche is differentially affected by the prenatal ZIKV infection compared to other sub-regions of the hippocampus and the cortex, an idea that has been posited in other models of immune activation [47].

It is now clear that ZIKV-induced congenital abnormalities extend well beyond microcephaly [48]. Our preliminary evidence of cerebrocortical dysplasia suggests that one or more steps involved in corticogenesis has been disrupted. Evidence in other ZIKV-infection models suggests that ZIKV preferentially infects neural progenitor cells [2,13,15,49], which are responsible for cellular differentiation, neuronal migration, and maturation of cortical structures in the brain [15]. Therefore, it is possible ZIKV may target neural progenitor cells or their function in our rat model and thus explain the dysplasia of cortical neurons observed here.

These important experiments provide the first indication that a rat model of maternal ZIKV infection is an effective model for the studying the effects of ZIKV infection on fetal brain development. Future experiments should examine the full translational capability of this model, and the mechanisms by which prenatal ZIKV infection disrupts brain development. We are confident that our rat model will be effective for studying the negative long-term consequences and outcomes of ZIKV infection on affected offspring with the goal of developing preventative or therapeutic measures for its devastating effects.
